# Extensive Disease of the Ear and Bones of the Head

**Published:** 1855-07

**Authors:** 


### ARTICLE XIII.

### *Extensive Disease of the Ear and Bones of the Head.*

Mr. Part, at the North "Medical Society," related the case of a clergyman, twenty-five years of age, who, for a period of five years, had suffered from purulent discharge from the right ear, attended occasionally with great pain; coming suddenly to a fatal termination without the extent of mischief having been suspected. The patient who was born in India, of English parents, came to England when eight years of age, had always been well fed and clothed, and had never been subject to mercurial treatment, having led a very regular life. He had lost a brother and sister from scrofulous diseases. About five years before his death, after taking cold and having two severe falls on his head about the same time, he was attacked with acute pain in the ear, followed by a copious offensive puriform discharge from the meatus, accompanied by loss of hearing. He consulted several eminent aurists for this affection, but did not derive any benefit from the treatment recommended. In the summer of 1849, he consulted Mr. Pilcher, who kindly allowed Mr. Part to see his notes of the case, which he pronounced to be acute otitis, with fungus of the right meatus. Under the care of this gentleman, he improved considerably in health, but was constantly taking cold and suffering from a recurrence of his symptoms. He then became subject to fits of giddiness, in which he usually fell down; these were succeeded by vomiting and great pain in the ear and head. A year since, two small glands behind the ear began to enlarge, and ultimately suppurated; since remaining, fistulous openings, discharging freely at times, when that from the ear diminished, and increasing when the latter subsided. He was compelled at this time to give up his clerical duties. During the last year he has taken sarsaparilla and iodine. When Mr. Part first saw the patient, on the 19th of

July, 1852, he was suffering from severe pain in the head, greatest at the back; vomiting; inability to move without excessive pain; and could only lie with the head perfectly horizontal to the body. The countenance heavy, and not symmetrical; partial ptosis, and turning up of the eyeballs; slight squinting, and the right angle of the mouth drawn up. There was a swelling in front of the right ear, extending nearly to the margin of the orbit; great tenderness of the concha, with copious puriform discharge from the external meatus. Behind this, two fistulous openings communicating with one another; skin cool, except on the forehead, which was very hot; hands and feet cool; pulse 68, and full; tongue coated, protruded towards the right side; considerable thirst; bowels constipated, had not acted for three days; urine scanty and high colored, with hesitation in passing it; answered slowly and imperfectly; manners strange, morose, and inconsistent. By acting on the bowels freely, and the administration of saline diaphoretics, a certain amount of amendment was obtained, which continued up to the 25th, six days from the first time he was seen by Mr. Part. As he had passed restless nights, suffering great pain, muriate of morphia was administered at night, which secured him sleep. On the 23rd as he was suffering intense pain, a large blister was applied to the nape of the neck, which gave great relief to the pain in the head. From the 25th the patient gradually became worse; he grew restless, continually desiring to change his room; his speech and deglutition became more and more impaired; he had difficulty in micturating, and at last complete retention, requiring the introduction of the catheter. The swelling in front of the ear having increased in size, an opening was made into it by Mr. B. Cooper, who was called in with Dr. Babington, but only a small quantity of pus was evacuated. The pain in the head and ear now became more severe, and the tongue more coated. There was now difficulty in rousing him. He could not be moved, and the evacuations were passed involuntarily in bed. On the 2nd of August slight tetanic symptoms set in, the eretor muscles of the spine assumed a state of opisthotonos, and he died on the morning of the 3rd of August, at four A. M.

*Post-mortem Examination, eight hours after death.*—On cutting through the integuments on the swelling in front of the ear a cavity as large as a hazel-nut was met with, and communicating with this was another beneath the temporal muscle, as large as a walnut. Both were filled with a soft, cheesy substance. A probe passed into the cavity struck against the dura mater lining the squamous bone. On opening the head the dura mater was found greatly injected outside, and pink within, and was entire and adherent over the whole surface of the temporal bone within the cavity of the skull, but thickened in the right middle lateral fossa. When it was removed, the whole of the petrous bone, the basilar process of the occipital as far back as the posterior third of the foramen ovale, and the larger wing of the sphenoid, extending onwards to the middle line of the skull, were ascertained to be degenerated into a soft, cheesy mass, similar to that contained in the cavities above mentioned. A probe entering the opening behind the ear passed easily until it appeared in the foramen ovale, and another passed into the meatus appeared completely through the petrous bone. The malar bone was entirely destroyed, and the mastoid process of the temporal was also completely occupied by disease. The ventricals contained three ounces of bloody serum; arachnoid much injected, and between it and the pia mater was a layer of very yellow pus extending along the base of the brain, greatest in quantity on the anterior surface of the medulla oblongata, and extending down in front of the medulla spinalis as far as a view could be obtained. In the middle lobe of the brain was an abscess containing upwards of an ounce of very fetid greenish pus, and a second abscess existed in the middle of the posterior lobe containing a similar kind of pus. The section of the cerebral substance presented an unusual number of bloody points. No similar deposition was found elsewhere in the body.

